# Beyond PSA: The Role of Prostate Health Index (phi)

**DOI:** 10.3390/ijms21041184

**Published:** 2020-02-11

**Authors:** Matteo Ferro, Ottavio De Cobelli, Giuseppe Lucarelli, Angelo Porreca, Gian Maria Busetto, Francesco Cantiello, Rocco Damiano, Riccardo Autorino, Gennaro Musi, Mihai Dorin Vartolomei, Matteo Muto, Daniela Terracciano

**Affiliations:** 1 Division of Urology, European Institute of Oncology, 20141 Milan, Italy; matteo.ferro@ieo.it (M.F.); ottavio.decobelli@ieo.it (O.D.C.); gennaro.musi@ieo.it (G.M.); 2 Department of Emergency and Organ Transplantation—Urology, Andrology and Kidney Transplantation Unit, University of Bari, 70124 Bari, Italy; pappt1@yahoo.it; 3 Department of Urology, Abano Terme Hospital, 35031 Padua, Italy; info@angeloporreca.it; 4 Department of Urology, Sapienza University of Rome, 00185 Roma, Italy; gianmaria.busetto@uniroma1.it; 5 Department of Urology, Magna Graecia University of Catanzaro, 88100 Catanzaro, Italy; francesco.cantiello@unicz.it (F.C.); rocco.damiano@unicz.it (R.D.); 6 Division of Urology, VCU Health System, Richmond, VA 23219, USA; ricautor@gmail.com; 7 Department of Urology, Comprehensive Cancer Center, Vienna General Hospital, Medical University of Vienna, Währinger Gürtel 18–20, 1090 Vienna, Austria; 8 Department of Cell and Molecular Biology, University of Medicine, Pharmacy, Sciences and Technology, 540139 Targu Mures, Romania; 9 Radiotherapy Unit, “S. G. Moscati” Hospital, 83100 Avellino, Italy; matteo.muto@unina.it; 10 Department of Translational Medical Sciences, University of Naples “Federico II”, 8031 Naples, Italy

**Keywords:** phi, prostate cancer, overdiagnosis, clinical significance

## Abstract

Background: Widespread use of prostate specific antigen (PSA) in screening procedures allowed early identification of an increasing number of prostate cancers (PCas), mainly including indolent cancer. Availability of different therapeutic strategies which have a very different impact on the patient’s quality of life suggested a strong need for tools able to identify clinically significant cancer at diagnosis. Multi-parametric magnetic resonance showed very good performance in pre-biopsy diagnosis. However, it is an expensive tool and requires an experienced radiologist. In this context, a simple blood-based test is worth investigating. In this context, researchers focused their attention on the development of a laboratory test able to minimize overdiagnosis without losing the identification of aggressive tumors. Results: Recent literature data on PCa biomarkers revealed a clear tendency towards the use of panels of biomarkers or a combination of biomarkers and clinical variables. Phi, the 4Kscore, and Stockholm3 as circulating biomarkers and the Mi-prostate score, Exo DX Prostate, and Select MD-X as urinary biomarker-based tests have been developed. In this scenario, phi is worthy of attention as a noninvasive test significantly associated with aggressive PCa. Conclusions: Literature data showed that phi had good diagnostic performance to identify clinically significant (cs) PCa, suggesting that it could be a useful tool for personalized treatment decision-making. In this review, phi potentialities, limitations, and comparisons with other blood- and urinary-based tests were explored.

## 1. Background

Overdiagnosis and overtreatment are well-recognized consequences of widespread PSA use in screening procedures. In 2014, Richard Ablin, who discovered PSA in 1970, published a book entitled “The Great Prostate Hoax” (Published March 4th, 2014 by St. Martin’s Press), talking about public health disasters and highlighting the two main limits of PSA: it is not cancer-specific and it does not distinguish indolent and aggressive cancer [1]. The latter is an essential criterion for a biomarker in the clinical scenario of PCa, characterized by a marked variability of disease behavior and by the diversity of available treatment and related impairment of quality of life. In this context, there is a strong need for a personalized approach in which biomarkers might play a relevant role. On this basis, researchers’ attention focused on the development of a laboratory test able to minimize overdiagnosis without missing the target in order to provide clinicians information useful to choose the best treatment for each patient: the one that matches tumor aggressiveness and therapy invasiveness [2].

To address this issue, many new tests have been proposed, based either on circulating and urinary biomarkers, with a clear tendency towards the use of panels of biomarkers, or a combination of biomarkers and clinical variables (Table 1), in an attempt to overcome the inadequacy of a single biomarker, learned by the lesson of PSA [3,4].

Among next-generation PCa tests, phi is the one for which more scientific evidence is available.

The aim of this review is to discuss the state-of-the-art of the role of phi in the detection of clinically significant prostate cancer (csPCa) and its application in contemporary practice. Furthermore, we evaluated diagnostic pathways that combine phi with other stratification tools such as multi-parametric magnetic resonance imaging (mp-MRI) to potentially realize a high csPCa detection rate together with high cost-effectiveness.

## 2. The Emerging Role of Phi

Phi, the 4Kscore, and S3M are multiplex tests including different molecular forms of PSA. In 1990, for the first time, free and complexed PSA were differentiated [10]. In 2000, Mikolajczyk [11] characterized different components of free and complexed PSA. PSA is a serine protease that physiologically dissolve seminal clots, degrading semenogeline and fibronectin. PSA is synthesized as an inactive precursor, preproPSA; a 17-aa cut at the amino-terminal end produces proPSA, still an inactive precursor, which is released in the prostate lumen. The 7-aa cut at the aminoterminal end of proPSA generates mature enzyme, which enters the blood and is bound by protease inhibitors, especially α1-antichymotripsin. Inactive fragments from proteolytic degradation of the mature enzyme represent free PSA (fPSA), together with the precursor, pro-PSA. The isoform [-2]pro-PSA is preferentially synthesized in malignant cells [5,12,13,14]. Malignancy leads to an alteration of tissue architecture and less efficiency of this complex series of enzymatic reactions. Consequently, there is an increase in circulating levels of complexed PSA (cPSA) and pro-PSA and a decrease of fPSA [12]. Accordingly, for many years, a low f/totalPSA (f/tPSA) ratio has been used to guide diagnosis in patients with PSA values in the “grey” zone [15].

Literature data indicated that a f/tPSA ratio lower than the cut-off value is associated with malignancy in only about 42% of cases [16]. Therefore, performing a biopsy on a patient with PSA values in the “grey zone” on the basis of the f/tPSA ratio is the same as throwing a coin.

In an attempt to improve the poor diagnostic performance of the f/tPSA ratio, other PSA molecular forms have been studied; in particular, [-2]proPSA, whose production is selectively increased in cancer and is significantly associated with high-grade cancer (Gleason score ≥ 7) at radical prostatectomy [13,14]. Based on these findings, phi was developed. Phi is a multifactorial mathematical combination of PSA, fPSA, and [-2]proPSA, which produces a risk index of positive biopsy. Intuitively, a higher [-2]proPSA/fPSA ratio associated with high tPSA may suggest a diagnosis of clinically significant PCa [5].

Phi was approved by the FDA in 2012 for men over 50 years of age, with negative DRE and PSA in the grey zone (between 4 and 10 ng/mL).

Following FDA approval, a lot of studies have focused on the comparison between the diagnostic performance of phi and the f/tPSA ratio.

## 3. Biopsy

Catalona et al. [14] reported the results of a multi-center study on 892 men undergoing first (79%) and repeat biopsy. Phi values significantly outperformed the f/t PSA ratio specificity for PCa detection in men with PSA in the 2 to 10 ng/mL range and negative DRE. Moreover, the authors demonstrated that high phi values were significantly associated with high-grade cancer at biopsy (*p* = 0.01).

These findings were later confirmed by a study [17] on 658 men with PSA values between 4 and 10 ng/mL, showing that phi is the best predictor of csPCa, based not only on the Gleason score (Gs), but also on Epstein criteria (AUC phi 0.707 for Gs ≥ 7 and 0.698 for Epstein criteria versus 0.66 and 0.65 %fPSA, respectively). This last evidence is particularly interesting since these criteria are used to select patients for active surveillance (AS), suggesting that phi could be useful as a tool to choose the best therapeutic option. In a prospective study on 251 subjects at first biopsy, we demonstrated that phi outperformed PSA and the fPSA/tPSA ratio in terms of the ability to predict positive biopsy [18].

A meta-analysis in 2014 demonstrated that the probability of identifying a positive biopsy significantly increased using phi in place of the free/total PSA ratio in subjects with PSA ranging from 2 to 10 ng /mL [19].

## 4. Active Surveillance

In a study including 300 patients at first biopsy, we demonstrated that lower phi values were significantly associated with low-risk cancer eligible for AS [20].

In addition, there is evidence that phi is able to predict biopsy reclassification in men under AS. In a study involving 167 men undergoing an active surveillance protocol at Johns Hopkins University [21], the authors showed that phi values were significantly associated with biopsy reclassification (37 vs. 27, *p* = 0.002), in particular, either the basal and longitudinal phi measure was significantly associated with biopsy reclassification defined on the basis of disease extension [(prostate volume (*p* = 0.049), biopsy core number (*p* = 0.047), and the percentage of positive cores (*p* = 0.023)] and of Gs upgrading. These data were later reproduced in a Japanese study [22] including 118 subjects on AS. The authors showed that phi was a significant independent predictor of biopsy reclassification one year after entering AS (*p* = 0.008).

The ability of phi to predict csPCa was well-summarized in the meta-analysis [23], including 16 published studies and more than 6000 patients. The authors evaluated phi for the detection of high-grade (Gs ≥ 7) PCa and reported a high diagnostic accuracy (pooled sensitivity of 0.90, pooled specificity of 0.17). These findings suggested that, if routinely used, phi would reduce the rate of unnecessary biopsies, without missing aggressive PCa. Such an ability was markedly highlighted in a Prometheus study [24], involving 646 patients with PSA values between 2 and 10 ng/mL and PCa family history. The authors reported that phi was able to miss a significantly lower percentage of high-grade cancer (Gs ≥ 7) compared to %fPSA (1.1% vs. 7.5%).

More recently, a multicentric study [25] including 1652 subjects (503 European and 1149 Asian) showed that using PHI, more biopsies could be avoided in Asian than in European men (56% vs. 40%). This finding was not surprising, taking into account that an Asian man had a PCa risk about four-fold lower than a European man. Moreover, the results of this study suggested the need for different phi reference ranges in men of different ethnic groups. In particular, this study recommended phi > 30 to predict high-grade (Gs ≥ 7) cancer in Asian men, whereas the threshold should be >40 for European men.

## 5. PHI-Density (PHID)

Recently, the ability of PHID to identify csPCa has been studied. In a prospective study on 118 men on first biopsy, higher PHID (calculated as phi divided by prostate volume as determined by the trans-rectal ultra-sound ratio) was significantly associated with the prevalence of csPCa [26]. ROC curve analysis of univariable logistic regression models predicting csPCA showed that PHID outperforms PHI (AUC 0.84, 95%CI 0.77–0.91 versus 0.76, 95%CI 0.68–0.85) [26].

Druskin et al. [27] evaluated the combination of PHID (retrospectively calculated as phi divided by prostate volume measured at biopsy) and multi-parametric MRI (mp-MRI) in 104 subjects: Pi-RADS ≥ 3 combined with PHID ≥ 0.44 identified nearly 100% csPCa.

However, PHID has not been validated, and when prostate volume is calculated at biopsy, it cannot be used as a tool to select men who need biopsy.

## 6. Multi-Parametric MRI (mp-MRI)

In the last few years, mp-MRI has shown growing relevance in pre-biopsy diagnosis of PCa [28]. In this context, it could be interesting to evaluate how phi integrates with mpMRI. Gnanapragasam et al. [29] first reported in a repeat biopsy population in which adding phi to mp-MRI (mpMRI-1.5T MR450 or the 3T Discovery MR750-HDx system) improved predictive accuracy of csPCa (AUC 0.75 vs. 0.64). Moreover, DCA showed that the addition of phi to mp-MRI allowed the avoidance of more unnecessary biopsies, missing a very low number of high-grade cancers (1 versus 21 using only mp-MRI). Worthy of attention, phi values were generally higher in patients with an mpMRI lesion, but the use of phi in place of PSA only slightly increased predictive accuracy, for any Likert lesion (AUC 0.60 [0.53–0.67] vs. 0.47 [0.40–0.54]) and a Likert 4/5 lesion (AUC 0.59 [0.52–0.66] vs. 0.51 [0.44–0.58] for PSA). These findings suggest that the phi has a complementary role with mp-MRI, but it is not a test useful to predict if an mpMRI lesion will be present.

Hsieh et al. [30] confirmed that adding phi to mp-MRI improved the predictive accuracy and clinical net benefit for 102 Asian subjects undergoing first biopsy. ROC curve analysis for the identification of csPCa showed that AUC of the combination phi-imaging was significantly higher (0.87) than phi alone (0.73, *p* = 0.002) and mp-MRI alone (0.83, *p* = 0.035). Moreover, adding phi to mp-MRI provided an increased ability to reduce unnecessary biopsies: up to 50% could be avoided if only patients with PI-RADS 3–5 and phi ≥ 30 were selected. Worthy of particular interest were the results obtained by Tosoian et al. [26] at Johns Hopkins University. The authors showed that phi was able to identify csPCa in patients with PI-RADS 1 (corresponding to a benign condition) and 3 (which is an intermediate score). These findings suggested that phi could be useful to select patients with negative or inconclusive imaging for biopsy.

A plausible scenario will be that subjects with PI-RADS 5 lesions should proceed to biopsy, whereas for PI-RADS ≤ 4 lesions, phi should be determined to select patients who really need a biopsy. This could be particularly relevant since literature data demonstrated a wide range of negative predictive values (NPV) for csPCa identification (63%–98%) [31], leading to a high rate of repeated unnecessary biopsies in men with negative mpMRI. In such a context, a non-invasive test such as phi could be very useful to choose the patients who will benefit most from biopsy, avoiding the risk for patients and the waste of money.

## 7. Radical Prostatectomy

The best strategy to evaluate phi association with PCa aggressiveness is to evaluate the correlation between the phi pre-operative level and definitive histology obtained after radical prostatectomy. Several studies showed a significant association between phi values and unfavorable prognostic features defined at surgery, as the pathological grade and stage and tumor volume.

Guazzoni et al. [32] showed in a prospective observational study on 350 subjects that phi levels were significantly higher in patients with T3, Gs ≥ 7, and Gleason upgrading (*p* < 0.0001) and significantly lower in subjects with tumor volume <0.5 (*p* < 0.0001). We found that phi was an accurate predictor of advanced stage and high-grade at RP [33]. The significant association with the pathological stage (*p* = 0.001) and grade (*p* = 0.002) was later confirmed in a very large population (more than 1600 subjects) by Tosoian et al. [34]. In addition, Cantiello et al. showed that phi outperformed PCA3 in the ability to predict Gs ≥ 7 [35]. Several authors demonstrated that phi was a good predictor of local tumor extension in GS 6 cancers and has a better predictive accuracy of RP pathological outcomes compared to currently used biomarkers [36,37,38].

In a recently published study [39], the authors showed that phi is the most accurate predictor (AUC 0.74 [0.68–0.80] of definitive Gs higher than 6. This is particularly relevant since a Gs ≤ 6 is one of the criterion used for the selection of patients for AS, suggesting that phi could be advantageous to choose the best treatment option.

## 8. Biochemical Recurrence (BCR)

Further evidence supporting the association between phi values and PCa aggressiveness is available from studies which evaluated the ability of phi to predict BCR. Lughezzani et al. [40] demonstrated that high preoperative phi values were significantly associated with shorter BCR-free survival at 2 years (*p* < 0.001) in a study population including 313 subjects treated with robotic-assisted radical prostatectomy. The authors reported a BCR-free survival rate of 69.7% for subjects with pre-operative phi levels ≥ 82 versus 97.7% for patients with lower values. These findings suggest that phi could be informative to evaluate the need for adjuvant therapy and the follow-up schedule after surgery.

Recently, Maxeiner et al. [41] showed in a study population of 437 patients with a longer than 5-year follow-up that phi is the most accurate predictor of BCR with an AUC of 0.623 [0.559–0.688] versus PSA of 0.59 (*p* < 0.001).

## 9. Comparison with Other Tests Based on Circulating Biomarkers

Collectively, literature data indicated that phi is potentially useful to reduce the number of unnecessary biopsies and to ameliorate the identification of csPCa [23]. On this basis, since 2015, phi was recommended by the European Association of Urology to reduce the number of unnecessary prostate biopsies in PSA-tested men, to improve prediction of csPCa, in men with a PSA between 4–10 ng /mL and between 2–10 ng /mL, and phi also has a role in monitoring men under active surveillance (https://www.scribd.com/document/376891488/2018-edition-of-the-european-association-of-urology-eau-guidelines).

In 2018, the 4K score was included in EAU guidelines. This test combines the value of four kallikreins (PSA, fPSA, iPSA, and hk2) with clinical variables such as age, DRE, and previous biopsy result to obtain a risk index indicating whether the patient has an aggressive tumor. Some authors demonstrated that the 4Kscore is significantly associated with the presence of distant metastases [6,42]. Moreover, the 4Kscore showed a performance comparable to phi both for the identification of positive biopsies (AUC = 0.69 [0.64–0.73] versus 0.70 [0.66–0.75], *p* < 0.0001) and of high-grade tumors (AUC = 0.72 [0.67-0.77] versus 0.71 [0.66–0.76], *p* < 0.0001) [43]. These findings have been confirmed by a meta-analysis including 16,762 patients showing that phi and the 4KScore were comparable in their ability to detect overall and high-grade PCa [44].

More recently, the S3M test was developed at the Karolinska Institute in Stockholm [45]. S3M combines several clinical variables (age, family history, previous biopsies, prostate volume, DRE) with 232 single nucleotide polymorphisms (i.e., IL-4, MGMT, AKT) and 6 protein markers (PSA, fPSA, iPSA, hk2, MSMB, and MIC1 [45]. S3M was validated on 60000 subjects, showing an AUC significantly higher than PSA (AUC = 0.75 [0.73–0.77] versus 0.58 [0.57–0.60]) for the identification of high-grade tumors [46]. Of note, in a study including 532 Scandinavian patients at first biopsy, using S3M to select patients for MRI and targeted biopsy prevented 42% of biopsies, decreasing detection of indolent cancer, and maintaining a good sensitivity to identify csPCa [47].

Currently, a head-to-head comparison of S3M with phi is still lacking.

Based on literature data (Table 2), phi, the 4Kscore, and S3M seem to show comparable diagnostic performance for the identification of csPCa. Phi has the lowest cost and it is the only FDA approved method and with a CE mark. Conversely, S3M is available for clinical use only in Sweden, being validated on a large population, but homogeneous by ethnicity, mainly northern European men.

Finally, another test based on circulating biomarkers has been developed called iXip. It is a risk index of positive biopsy calculated using an algorithm which combines PSA, the PSA-IgM immunocomplex, and prostate volume. Few literature data are available for iXip compared with PHI, the 4K score, and S3M. However, two studies are worthy of interest. The first is a multicentric study including 426 patients who were candidates for first biopsy showing that iXip values were significantly higher in PCa patients and iXip values lower than 20% corresponded to 0 patients with PCa. In this study, the authors also showed a significant correlation between iXip and biopsy Gs [48].

These findings were confirmed in a study by Galosi et al. [49] on 219 men who were candidates for re-biopsy.

At present, iXip seems to be promising, but the literature still lacks data on the association with PCa aggressiveness defined at radical prostatectomy and with criteria used to select patients for AS. Moreover, head-to-head comparisons with PHI, the 4K score, and S3M are not available.

## 10. Comparison with Other Tests Based on Urinary Biomarkers

In the last ten years, numerous urinary tests have also been developed, with a clear tendency to combine multiple markers. Historically, the first 2 urinary markers proposed for PCa were PCA3 (prostate cancer antigen 3) and the T2: ERG fusion gene.

PCA3 is a long-noncoding RNA overexpressed in PCa. Its overexpression involves the down-regulation of the PRUNE2 onco-suppressor gene and the dysregulation of proteins involved in the epithelial-mesenchymal transition such as vimentin and E-cadherin. The fusion of T2 with the ERG gene, present in about 2/3 of the newly diagnosed cases, entails the overexpression of several genes involved in tumor progression [50]. For both markers, post-DRE urine tests were developed [51].

The PCA3 score is significantly associated with the presence of positive biopsy. A recent meta-analysis demonstrated good diagnostic performance of the marker [52].

We first compared phi with PCA3 in 151 patients at initial biopsy, showing that phi and PCA3 were accurate predictors of cancer (AUC 0.77 and 0.71, respectively). There was no significant difference from PCA3 (*p* = 0.368), suggesting a comparable ability to discriminate benign and malignant conditions [18].

In a second study, PCA3 showed a slightly lower AUC compared to phi to predict positive first biopsy. However, based on DCA (decision curve analysis) that provided the clinical net benefit of a test, phi compared to PCA3 prevents a higher number of unnecessary biopsies without losing the identification of high-grade cancer. The combination of phi and PCA3 was able to significantly improve cancer identification compared to only one biomarker [20]. Scattoni et al. [53] demonstrated that phi AUC (0.70, 95% CI: 0.63–0.76) was higher than PCA3 (AUC 0.59, 95% CI: 0.52–0.66; *p* = 0.043). Regarding the ability to predict pathological features at radical prostatectomy, Cantiello et al. [54] showed that the addition of phi to a base model (including age, total PSA, fPSA, rate of positive cores, clinical stage, prostate volume, body mass index, and biopsy Gs) significantly improved the ability to predict pathologically confirmed significant PCa. We found that Phi outperforms PCA3 in the ability to predict TV ≥ 0.5 mL (0.94 vs. 0.86), Gs ≥ 7 (0.94 vs. 0.78), and tumor stage (0.85 vs. 0.74) [33].

Decision curve analysis showed that phi had a higher net benefit compared to PCA3 in the improvement of patient selection for AS using both Epstein and PRIAS criteria [35].

Of note, PCA3 showed a controversial correlation with aggressiveness. Auprich et al. showed that PCA3 was significantly associated only with some of the characteristics of tumor aggressiveness such as the tumor volume, Gleason score and pathological stage, but not with the presence of extracapsular extension or the invasion of the seminal vesicles [55]. Shalken and coworkers previously performed a similar study in a cohort of 70 patients, but the same significant correlation with tumor volume and with the Gs and pathological stage was not demonstrated [56]. In international guidelines, PCA3 is recommended only for patients undergoing repeated biopsy (https://www.scribd.com/document/376891488/2018-edition-of-the-european-association-of-urology-eau-guidelines).

In a prospective multicenter study performed on 516 patients, T2: ERG significantly correlated with the absence of malignancy, but not with the aggressiveness [57].

Furthermore, T2: ERG and PCA3 present a reduced clinical benefit in African–American subjects, suggesting that population composition could represent a relevant bias when the diagnostic performance of the two tests is evaluated [58].

PCA3 and T2: ERG have been combined into 2 different multiplex tests: the Mi-prostate score (MiPS) and ExoDx Prostate.

MiPS was developed at the University of Michigan and it combines PCA3 and T2: ERG with serum PSA, providing a score with good diagnostic performance for the identification of high-grade tumors [8].

ExoDx Prostate requires the determination of PCA3 and T2:ERG on the exosomes isolated from the patient’s urine [9,50]. This test shows a good AUC, which is further increased when PCA3 and T2:ERG are combined with the standards of care, i.e., PSA and age [9,50]. However, the pre-analytic phase of ExoDx Prostate remains rather complicated as it need the isolation of urinary exosomes.

Finally, SelectMDX was recently developed, which measures the expression of HOXC6 and DLX1 genes in post-DRE urine, providing a score significantly correlated with the probability of identifying a high-grade tumor [50]. In a large multicenter study, the addition of the score to the PSAD alone or to the PSAD and DRE led to an AUC of about 0.90 for the identification of high-grade tumors [7].

Among the urinary tests, PCA3 is recommended for patients who are candidates for repeated biopsy probably due to its controversial correlation with aggressiveness. Among multiplex tests, Select MDx is very promising, having a better diagnostic performance for the identification of clinically significant tumors and a lower cost. In the latest guidelines of the EAU, PCA3 is recommended for patients undergoing repeated biopsy and SelectMdx to identify the risk of a positive biopsy for high-grade cancer (https://www.scribd.com/document/376891488/2018-edition-of-the-european-association-of-urology-eau-guidelines).

## 11. Limitations

For several years, phi use in clinical practice was encouraged both to reduce unnecessary biopsies and to identify potential life-threatening cancers [59]. Nevertheless, phi does not have de facto widespread use, and this is partly due to two critical aspects.

The first one is the pre-analytical stability of [-2]proPSA. It is recommended that serum is separated within 3 h from blood draw, a requirement not simple to address in laboratory routine practice [60]. Semjonow et al. [60] demonstrated that circulating [-2]proPSA concentration increased over time following clotting. In whole blood at room temperature, a median phi increase not higher than 15% in the first 3 h has been reported [61]. Interestingly, in a recent study, Dittadi et al. [62] demonstrated that [-2]proPSA was more stable in plasma EDTA compared to serum. However, due to the limited number of cases examined in this study (*n* = 26), it is not still possible to assess whether the use of plasma EDTA improved phi clinical performance.

The second relevant limitation is the cost. Several studies simulated the impact of phi use on the total cost of diagnostic and therapeutic procedures for PCa patients [63,64,65,66,67].

Nichol et al. showed that adding phi in a 1-year health plan model with 100,000 men aged 50–74 years led to an expected cost for PCa diagnosis that was $356 647 lower than that using a tPSA threshold of ≥2 [67].

A budget impact analysis demonstrated that combining phi with tPSA in an annual screening cycle for prostate biopsy provided a cost saving of $1199 per person considering a tPSA threshold of ≥2 [66].

Collectively, these studies indicated that phi cost should be compensated by the cost of unnecessary biopsies and invasive treatment with their related complications avoided in indolent PCa.

## 12. Conclusions

At present, available literature data suggest that phi: (a) improves individual risk assessment for early PCa detection, (b) reduces unnecessary negative initial or repeated biopsies, (c) is significantly associated with clinically significant prostate cancer, (d) ameliorates the identification of insignificant PCa for AS, and (e) may play a role in treatment decision strategies.

Based on different fields of application of phi (Table 3), it could be interesting to consider whether this test might play a role as a tool able to diminish overdiagnosis and overtreatment in a PCa clinical scenario.

An improvement in the diagnosis of csPCa has been reached by mp-MRI, but with increased costs [28]. Taking into account the growing use of imaging, it is conceivable that novel biomarkers alone or in addition to risk calculators could be offered to men with suspicion of PCa (elevated PSA, abnormal DRE) in order to select patient that could take advantage of expensive tools such as mp-MRI and/or an invasive procedure such as a biopsy. Further to this point, phi is very promising since there is evidence that the addition of phi to a PCa risk calculator such as ERSPC and PCPT improves their ability to identify csPCa in men with PSA between 2 and 10 ng/mL (*p* < 0.05) [69,70]. At present, this multivariable approach seems to be the most likely for a personalized treatment choice, ensuring the best result for the patient in terms of survival as well as quality of life.

In the next years, as a simple blood test, the cost of phi will probably decrease, and it will be realistic that phi will play a pivotal role as a risk stratification tool in PCa diagnosis.

## Figures and Tables

**Table 1:** Next-generation prostate cancer tests: tendency towards the use of a combination of biomarkers or biomarkers and clinical variables.

Test	Variables	Reference
BLOOD-BASED		
Phi	[-2]proPSA, freePSA, PSA	[5]
4Kscore	Age, DRE, prior negative biopsy status, PSA, freePSA, intactPSA, hK2	[6]
Stockholm 3 (S3M)	Age, Family history, Previous biopsy, Prostate volume, DRE, PSA, fPSA, iPSA, HK2, beta-microseminoprotein, macrophage inhibitory cytokine, 232 SNP	[7]
URINE-BASED		
MI-Prostate score	Serum PSA, PCA3, TMPRSS2-ERG	[8]
Exo-Dx	Urine PCA3, TMPRSS2-ERG exosome expression	[9]
Select-MDX	Age, PSA, Prostate volume, Family history, DRE, DLX1, HOXC6, KLK3,	[7]

DRE: digital rectal examination, SNP: single nucleotide polymorphism, HK2: human kallikrein 2, PCA3: prostate cancer antigen 3, MiPS: Mi-Prostate score, TMPRRS2: transmembrane protease, serine 2.

**Table 2:** Comparison between phi and other blood-based laboratory tests.

	Phi	4K Score	S3M
AUC * ranges reported for csPCa	0.66–0.76	0.67–0.77	0.73–0.77
Cost	120 €	300 €	Estimated > 200 €
Calculated as	Mathematical Model	Algorithm	Algorithm
Availability	FDA-approved and CE-mark	CLIA-approved	For clinical use only in Sweden

* Area under ROC curve.

**Table 3:** Phi in PCa patients’ clinical management.

Outcome	Authors, Year	Sample Size	Study Design	Reference
Detecting PCa at biopsy	Catalona et al., 2011	892	Prospective, multicenter	[68]
	Ferro et al., 2012	251	prospective	[18]
	Loeb et al., 2015	658	Prospective, multicenter	[17]
	Chiu et al., 2019	1652	Prospective, multicenter	[25]
Predicting aggressiveness at RP	Guazzoni, 2012	350	prospective	[32]
	Ferro, 2015	78	prospective	[33]
	Cantiello, 2016	188	retrospective	[35]
	Tosoian, 2017	1663	prospective	[26]
	Dolejsova, 2018	320	prospective	[39]
Selecting and reclassifying patients for AS	Tosoian, 2012	167	Follow-up AS	[21]
	Ferro, 2013	300	prospective	[20]
	Hirama, 2015	118	Follow-up AS	[22]
Predicting BCR	Lughezzani, 2015	313	Follow-up after RARP	[40]
	Maxeiner, 2017	437	Follow-up after RP	[41]

RP, radical prostatectomy, AS, active surveillance, BCR, biochemical recurrence, RARP, robotic-assisted radical prostatectomy.

## Abbreviations

DRE	digital rectal examination
SNP	single nucleotide polymorphism
HK2	human kallikrein 2
PCA3	prostate cancer antigen 3
MiPS	Mi-Prostate Score
TMPRRS2	transmembrane protease, serine 2
AUC	area under curve
DCA	decision curve analysis
